# Ethnicity-based subgroup meta-analysis of the association of *LOXL1* polymorphisms with glaucoma

**Published:** 2010-02-06

**Authors:** Haoyu Chen, Li Jia Chen, Mingzhi Zhang, Weifeng Gong, Pancy Oi Sin Tam, Dennis Shun Chiu Lam, Chi Pui Pang

**Affiliations:** 1 Joint Shantou International Eye Center, Shantou University & the Chinese University of Hong Kong, Shantou, China; 2 Department of Ophthalmology and Visual Sciences, the Chinese University of Hong Kong, Hong Kong, China

## Abstract

**Purpose:**

To investigate the association and ethnic heterogeneity of lysyl oxidase-like 1 (*LOXL1*) single nucleotide polymorphisms (SNPs) with exfoliation syndrome (XFS)/exfoliation glaucoma (XFG) and other types of glaucoma.

**Methods:**

We performed meta-analysis and ethnicity-based subgroup analyses according to published studies. Allele and genotype frequencies of SNPs rs1048661, rs2165241, and rs3825942 were extracted for analysis in Reviewer Manager: (1) comparison of the allelic distributions between XFS and XFG, (2) allelic association of *LOXL1* SNPs with XFS/XFG, (3) associations in homozygote, heterozygote, and dominant and recessive models, and (4) allelic association with primary open angle glaucoma (POAG).

**Results:**

In total 24 reported articles were retrieved, including Caucasian, African, Japanese, Indian, and Chinese populations. There was no significant difference in the distributions of rs1048661, rs2165241, and rs3825942 between XFS and XFG. The G allele of rs3825942 was the common at-risk allele for XFS/XFG in all populations with a total odds ratio (OR) of 10.89. The total homozygote OR of rs3825942 was 9.06 for XFS/XFG combined, but the total heterozygote OR was not significant. We also found that in the recessive model, the total OR was 14.70. There was no association of the three SNPs with POAG.

**Conclusions:**

The association of rs3825942, but not rs2165241 or rs1048661, with XFS/XFG is consistent in different ethnic populations in the recessive model. *LOXL1* is not associated with POAG in all study populations.

## Introduction

Glaucoma is one of the leading causes of irreversible blindness worldwide. It is a group of heterogeneous diseases characterized by progressive loss of retinal ganglion cells and degenerative optic neuropathy with or without elevated intraocular pressure (IOP) [1]. Open angle glaucoma is a major form of glaucoma and can be classified into primary open angle glaucoma (POAG) and secondary open angle glaucoma. Exfoliation glaucoma (XFG) is the most common form of secondary open angle glaucoma and is caused by obstruction of extracellular material in the anterior chamber angle in exfoliation syndrome (XFS), which is an age-related disease characterized by deposits of extracellular material in the anterior segment, including the cornea, lens, iris, and anterior chamber [2]. The prevalence of XFS varies widely across different ethnic populations. In people aged 60 or above, the prevalence of XFS is 0.4% in Hong Kong Chinese [3], 0.7% in Singapore Chinese [4], 4.4% in Japanese [5], and 10%–20% in the Caucasian population [6].

The etiology and pathogenesis of glaucoma are complex and involve multiple genetic and environmental factors. Myocilin (*MYOC*) is a causative gene for POAG and cytochrome P450, 1B1 (*CYP1B1*) for congenital glaucoma [7,8]. Mutations in optineurin (*OPTN*) and WD repeat domain 36 (*WDR36*) do not directly lead to POAG but increase the risk [9,10]. As for XFS and XFG, significant association with three single nucleotide polymorphisms (SNPs), rs1048661, rs2165241, and rs3825942, in the *Lysyl oxidase-like 1* (*LOXL1*) gene was first discovered in 2007 in Icelandic and Swedish populations by a genome-wide association study [11]. SNP rs2165241 was marginally (p=0.04) associated with POAG in the Icelandic but not in the Swedish population, while rs3825942 and rs1048661 were not associated with POAG in either population [11].

Since then the association of *LOXL1* SNPs with XFS/XFG has been reported in Caucasian populations in the USA [12-16], Australia [17], Austria [18], Germany [19,20], Italy [20], and Finland [21] and in other ethnic groups, including Japanese [22-27], Indian [28], and Chinese [29,30]. *LOXL1* SNPs in POAG have also been studied in Caucasian [14,21,31], Japanese [22,24,27], Chinese [32], Indian [33], and African populations [31]. Other types of glaucoma, such as normal tension glaucoma and pigmentary glaucoma, have been investigated in India [33], Japan [27], Germany [19], and the USA [34]. Despite the large amount of information, the overall association of SNPs and the discrepancies between individual cohorts or subgroups remain to be characterized.

Meta-analysis is a very useful statistical tool not only to summarize data from individual studies concerning a specific research question but also to identify and analyze the consistency and discrepancies of individual studies or population subgroups. The prevalence of XFS/XFG varies widely among different ethnic populations mainly due to variations in genetic background. Therefore, to ascertain effects due to ethnic differences, meta-analysis of the genetic association of *LOXL1* SNPs with glaucoma could be performed based on subgrouping by ethnicity. In *LOXL1*, three SNPs (rs1048661, rs2165241, and rs3825942) were first found associated with XFS/XFG in Icelandic and Swedish populations. They appeared in most of the reported studies, despite some inconsistencies in their presence [16,24,29,31]. Several other SNPs at *LOXL1* (rs12437465, rs2304719, and rs3522) have also been reported [13,17,31], but their associations were due to intermarker linkage disequilibrium (LD) and limited functional studies of these SNPs were reported, so they were not included in this meta-analysis. In this study, we perform a subgroup based meta-analysis using all of the reported studies on the association of *LOXL1* SNPs (rs1048661, rs2165241, and rs3825942) with XFS, XFG, and POAG.

## Methods

An internet-based literature search was conducted on PubMed and Embase, using the search strategy (“LOXL1” OR “LOXL” OR “Lysyl oxidase-like”) AND (“glaucoma” OR “exfoliation” OR “pseudoexfoliation”). The cut-off date was December 12, 2009. In total 41 articles were obtained from PubMed and 38 from Embase. Articles meeting the following criteria were included for further data analysis: (1) case-control study, cohort study, or population-based epidemiological survey; (2) reports on the association of three SNPs in *LOXL1*, i.e., rs1048661, rs2165241, and/or rs3825942, with XFS, XFG, POAG, or/and other types of glaucoma; (3) studies with reported and accessible sample size, allele, and/or genotype frequencies/counts in both patients and controls; and (4) original research articles, not reviews or comments. Repetitive publications, which were ruled out through tracking references, were incorporated as the latest reports. If several different cohorts were reported in the same article, they were treated as independent studies.

The retrieved abstracts and full texts were separately reviewed by two investigators (H.Y.C. and W.F.G.) who also independently performed data extraction and quality evaluation. A third reviewer (L.J.C.) would participate in the review if there was disagreement in the retrieved information. Total consensus of the three reviewers needed to be obtained on all retrieved data. The ethnicity, sample size, age, sex, allele, and/or genotype frequency/counts in both patients and controls were recorded. For studies with no direct data of genotype or allele counts, we calculated the frequency according to the reported data, rounding to the closest integer. The allele counting was also calculated from genotype counting when needed.

The Review Manager (version 5.0.18; The Cochrane Collaboration, Copenhagen, Denmark and SPSS software (ver. 16.0; SPSS Inc., Chicago, IL) were used to perform the meta-analysis: (1) The Hardy–Weinberg equilibrium (HWE) of each SNP in the control group of each study was examined by using χ^2^ analysis; (2) meta-analysis of the *LOXL1* SNPs rs1048661, rs2165241, and rs3825942 was performed to compare the allelic distributions between XFS and XFG; (3) if there were no statistical differences in allelic distributions between XFS and XFG, these two groups were combined. Meta-analyses of the allelic associations of rs1048661, rs2165241, and rs3825942 with overall XFS and XFG were performed; (4) if positive results were obtained from allelic association analysis, genotypic association analyses were performed using different genetic models, including multiplicative models estimating the homozygous odds ratio (OR) and heterozygous OR as well as the dominant model and recessive model; and (5) the allele association of rs1048661, rs2165241, and rs3825942 with POAG was analyzed. All the meta-analyses were performed in subgroupings by ethnicity. In all of the meta-analyses, the ORs were estimated by the fixed or random model according to the heterogeneity test. When the heterogeneity test α was <0.1, a random model was applied, and when the heterogeneity test α was >0.1, a fixed model was applied. Sensitivity tests were performed, but studies with counting data that were computed from frequencies data were excluded.

## Results

There were 20 articles reporting the association of *LOXL1* SNPs with XFS/XFG in a total of 3,068 cases and 20,363 controls from 22 study cohorts (Table 1). Nine articles investigated the association between *LOXL1* SNPs and POAG in a total of 2,223 POAG patients and 16,664 controls from 13 cohorts (Table 2). The HWE of all three SNPs was tested in the control groups of all cohorts. No deviation from the HWE was identified.

**Table 1:** Characters of reported cohorts of pseudoexfoliation syndrome/exfoliation glaucoma association with *LOXL1* SNPs

**First author**	**Cohorts**	**Sample size**		**Age (years)**		**Sex (Male %)**		rs1048661 ** G%**		rs2165241 ** T%**		rs3825942 ** G%**		**Ref**
		**Case**	**Control**	**Case**	**Control**	**Case**	**Control**	**Case**	**Control**	**Case**	**Control**	**Case**	**Control**	
Aragon-Martin	American	287	333	NA	NA	NA	NA	84.3	70.3	73.4	44.8	95.9	79.8	[12]
Challa*	American	50	235	74.0±8.0	64.9±11.6	78.0	61.7	78.7	66.5	66.7	48.7	93.9	84.4	[13]
Fan	American	206	88	75	72	NA	43	82.9	71.9	76.0	45.6	98.8	79.5	[14]
Fingert	American	72	75	NA	NA	NA	NA	81.9	60.0	NA	NA	98.6	88.0	[15]
Yang	American	62	170	NA	74.2±8.8	38.7	52.9	NA	NA	83	53	100	85	[16]
Hewitt*	Australian	86	2087	76.4±8.1	68.6±10.0	37.2	44.8	78	66	NA	NA	95	84	[17]
Mossbock	Austrian	167	170	75.7	77.1	45.5	44.1	84.1	67.1	NA	NA	99.4	81.7	[18]
Wolf	Germany	128	280	71.9±9.7	66±13	44	41	84.4	66.0	78.2	49.1	99.2	85.6	[19]
Pasutto	Germany	517	348	76.6±8.5	73.9±6.4	44.5	43.4	81.8	64.4	75.2	48.2	95.1	85.7	[20]
Pasutto	Italian	209	70	78.3±7.7	75.2±7.4	39.2	35.7	82.5	69.3	79.8	51.5	100	82.1	[20]
Thorleifsson	Icelandian	130	14474	NA	NA	NA	NA	78.1	65.1	74.6	47.3	98.4	84.7	[11]
Thorleifsson	Swedish	199	198	NA	NA	NA	NA	83.4	68.2	81.3	53.5	99.5	87.9	[11]
Lemmela	Finnish	141	404	NA	NA	NA	NA	82.5	68.3	73.2	46.8	96.8	82.3	[21]
Ramprasad	Indian	52	97	68.9±11.4	64.1±7.2	51.9	53.6	72.1	63.4	NA	NA	92.3	74.2	[28]
Lee	Chinese	62	171	74.7±7.7	67.4±5.6	48.4	46.8	54.2	44.4	NA	NA	99.2	91.8	[29]
Chen	Chinese	50	124	70.4±7.6	63.8±5.1	62.0	57.6	11.0	48.0	2.0	10.0	100	90.0	[30]
Fuse	Japanese	56	138	74.8±6.2	68.0±7.7	55.4	55.1	3.6	49.3	1.8	5.8	100	87.7	[22]
Hayashi	Japanese	59	190	78.4±6.9	31.4±1.5	37.3	50.0	0.8	46.0	NA	NA	100	85.7	[23]
Mabuchi	Japanese	89	191	76.5±6.6	65.7±11.4	NA	NA	0.6	45.0	NA	NA	99.4	85.3	[24]
Mori	Japanese	95	190	75.7±8.1	65.0±6.8	NA	NA	0.5	47.4	NA	NA	99.5	85.0	[25]
Ozaki	Japanese	209	172	78.0±6.1	73.8±7.9	32.1	27.9	5.3	49.7	1.7	10.2	98.6	86.3	[26]
Tanito	Japanese	142	157	78.5±8.2	77.2±5.0	38.0	28.7	4.9	55.4	0.7	12.4	99.3	80.6	[27]

The asterisk indicates the counting data was calculated from rate data. NA: data not available. Ref: references.

**Table 2:** Characters of reported cohorts of primary open angle glaucoma association with *LOXL1* SNPs

**First author**	**Cohorts**	**Sample size**		**Age (years±SD)**		**Sex (Male %)**		rs1048661 ** G%**		rs2165241 ** T%**		rs3825942 ** G%**		**Ref**
		**Case**	**Control**	**Case**	**Control**	**Case**	**Control**	**Case**	**Control**	**Case**	**Control**	**Case**	**Control**	
Fan	American	331	88	75	72	NA	43.2	72.4	71.9	41.2	45.6	77.1	79.5	[14]
Thorleifsson	Icelandian	90	14474	NA	NA	NA	NA	71.1	65.1	55.0	47.3	87.2	84.7	[11]
Thorleifsson	Swedish	200	198	NA	NA	NA	NA	63.8	68.2	48.8	53.3	86.3	87.9	[11]
Lemmela	Finnish	71	404	NA	NA	NA	NA	69.4	68.3	45.7	46.8	78.7	82.3	[21]
Liu*	Caucasian	279	227	58.7±12.8	>55	NA	NA	NA	NA	42.4	48.6	82.9	84.4	[31]
Liu*	African American	193	97	55.3±13.3	>55	NA	NA	NA	NA	23.7	20.4	61.7	59.9	[31]
Liu*	Ghanaian	170	138	55.4±13.8	>55	NA	NA	NA	NA	22.6	19.3	62.2	57.0	[31]
Chakrabarti*	Indian	112	105	NA	NA	NA	NA	61.6	69.5	32.1	32.0	83.0	75.0	[33]
Fuse	Japanese	62	138	NA	68.0±7.7	NA	55.1	39.5	49.3	4.8	5.8	91.1	87.7	[22]
Mabuchi	Japanese	213	191	62.9±14.8	65.7±11.4	NA	NA	47.2	45.0	NA	NA	85.0	85.3	[25]
Tanito	Japanese	40	157	75.6±5.3	77.2±5.0	35.0	28.7	51.3	55.4	3.8	12.4	80.0	80.6	[27]
Gong	Southern Chinese	293	250	66.8±12.9	74.1±6.8	60.1	49.2	42.0	47.2	8.4	10.2	89.4	87.6	[32]
Gong	Northern Chinese	169	197	39.1±16.5	69.4±6.0	78.1	49.2	47.9	49.7	8.6	8.4	89.9	86.5	[32]

The asterisk indicates the counting data was estimated from frequency data. NA: data not available. Ref: references

**Figure 1:**
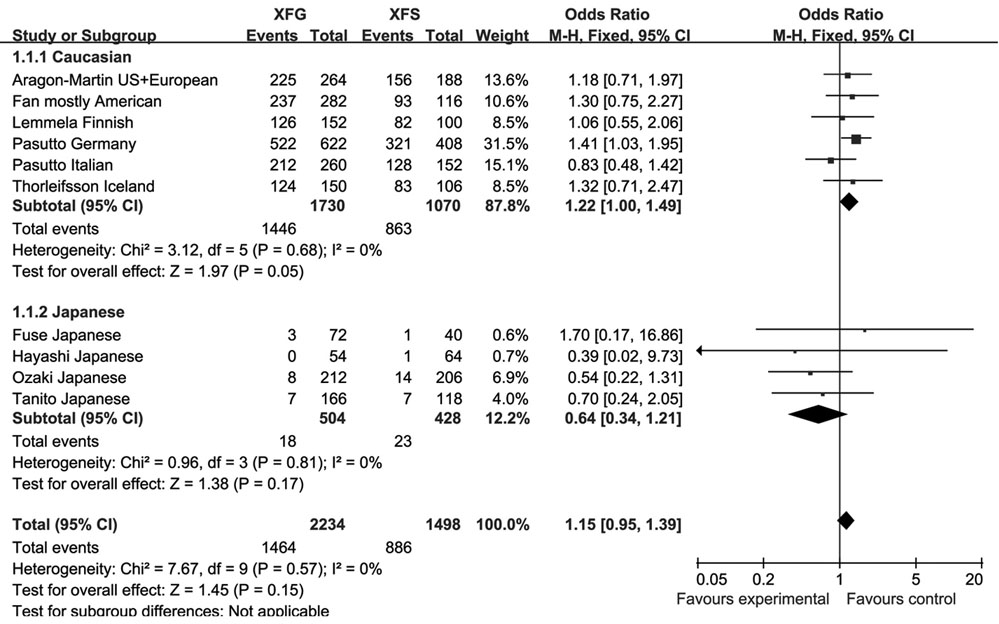
Meta-analysis of the distribution of single nucleotide polymorphism (SNP) rs1048661 between exfoliation syndrome (XFS) and exfoliation glaucoma (XFG). Squares indicate study-specific odds ratio (OR); the size of the box is proportional to the weight of the study; horizontal lines indicate 95% confidence interval (CI); diamond indicates summary OR with its corresponding 95% CI. Meta-analysis indicated there is no statistical difference of distribution of the SNP rs1048661 G and T allele between XFS and XFG.

**Figure 2:**
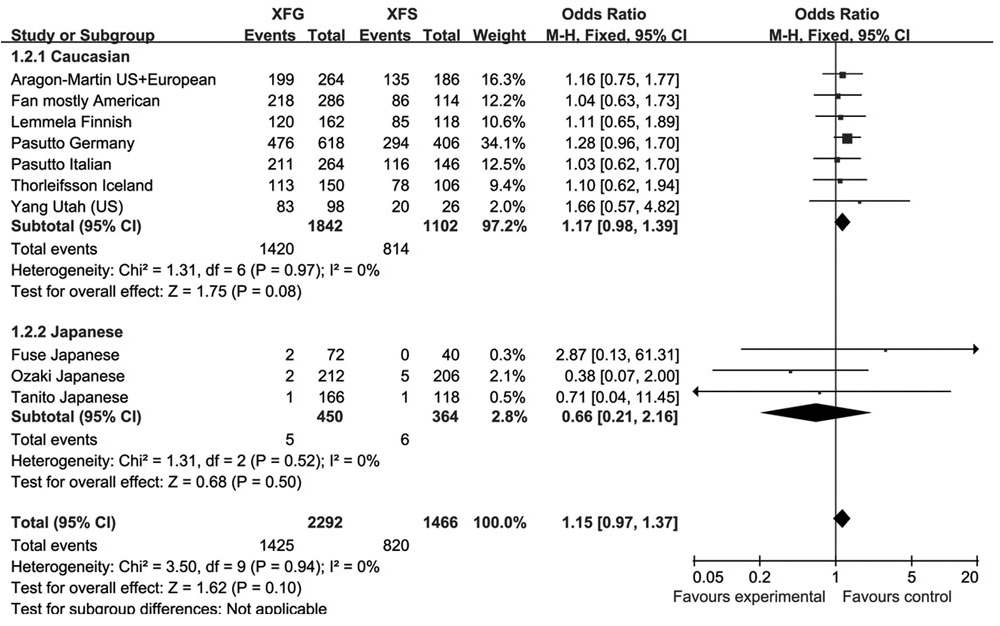
Meta-analysis of the distribution of single nucleotide polymorphism (SNP) rs2165241 between exfoliation syndrome (XFS) and exfoliation glaucoma (XFG). Squares indicate study-specific odds ratio (OR); the size of the box is proportional to the weight of the study; horizontal lines indicate 95% confidence interval (CI); diamond indicates summary OR with its corresponding 95% CI. Meta-analysis indicated there is no statistical difference of distribution of the SNP rs2165241 T and C allele between XFS and XFG.

**Figure 3:**
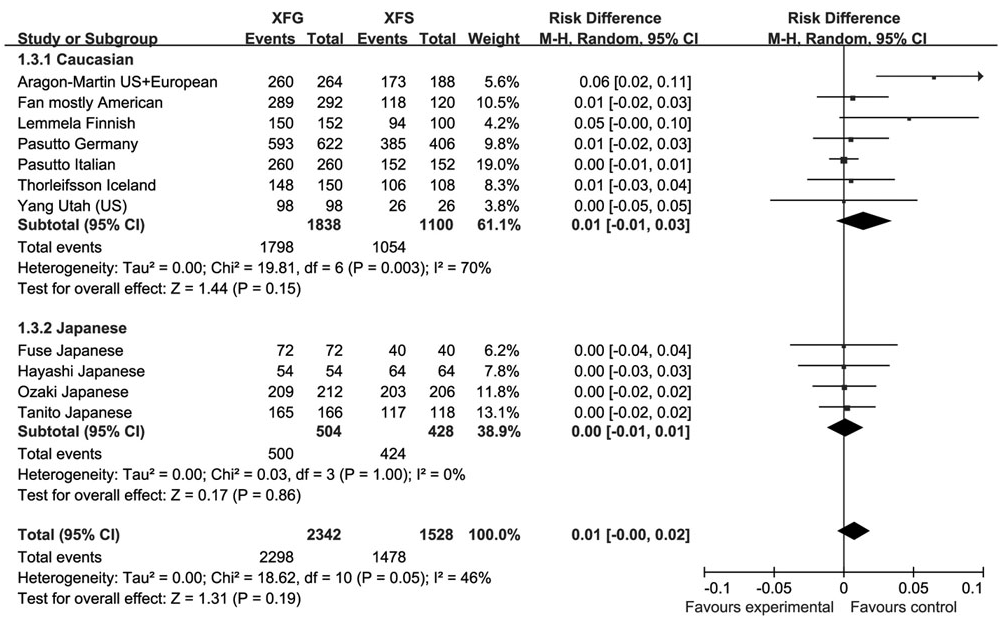
Meta-analysis of the distribution of single nucleotide polymorphism (SNP) rs3825942 between exfoliation syndrome (XFS) and exfoliation glaucoma (XFG). Squares indicate study-specific risk difference (RD); the size of the box is proportional to the weight of the study; horizontal lines indicate 95% confidence interval (CI); diamond indicates summary RD with its corresponding 95% CI. Meta-analysis indicated there is no statistical difference of distribution of the SNP rs3825942 G and A allele between XFS and XFG.

There was no significant difference in allele frequencies between XFS and XFG for rs1048661 and rs2165241 in any cohort. The overall OR for the two SNPs was 1.15 (95% confidence interval [CI] 0.95–1.39, p=0.15, Figure 1) and 1.15 (95% CI 0.97–1.37, p=0.10, Figure 2), respectively. The OR due to rs3825942 could not be estimated in four out of the 11 cohorts due to 100% G allele frequencies occurring in both the XFS and XFG groups. Therefore, we calculated the risk difference instead of the OR. The risk difference between XFS and XFG was 0.01 (95% CI −0.00–0.02, p=0.20, Figure 3), and showed no statistically significant difference in the distributional profiles of the three SNPs between XFS and XFG. We thus combined the XFS and XFG groups in subsequent analyses.

**Figure 4:**
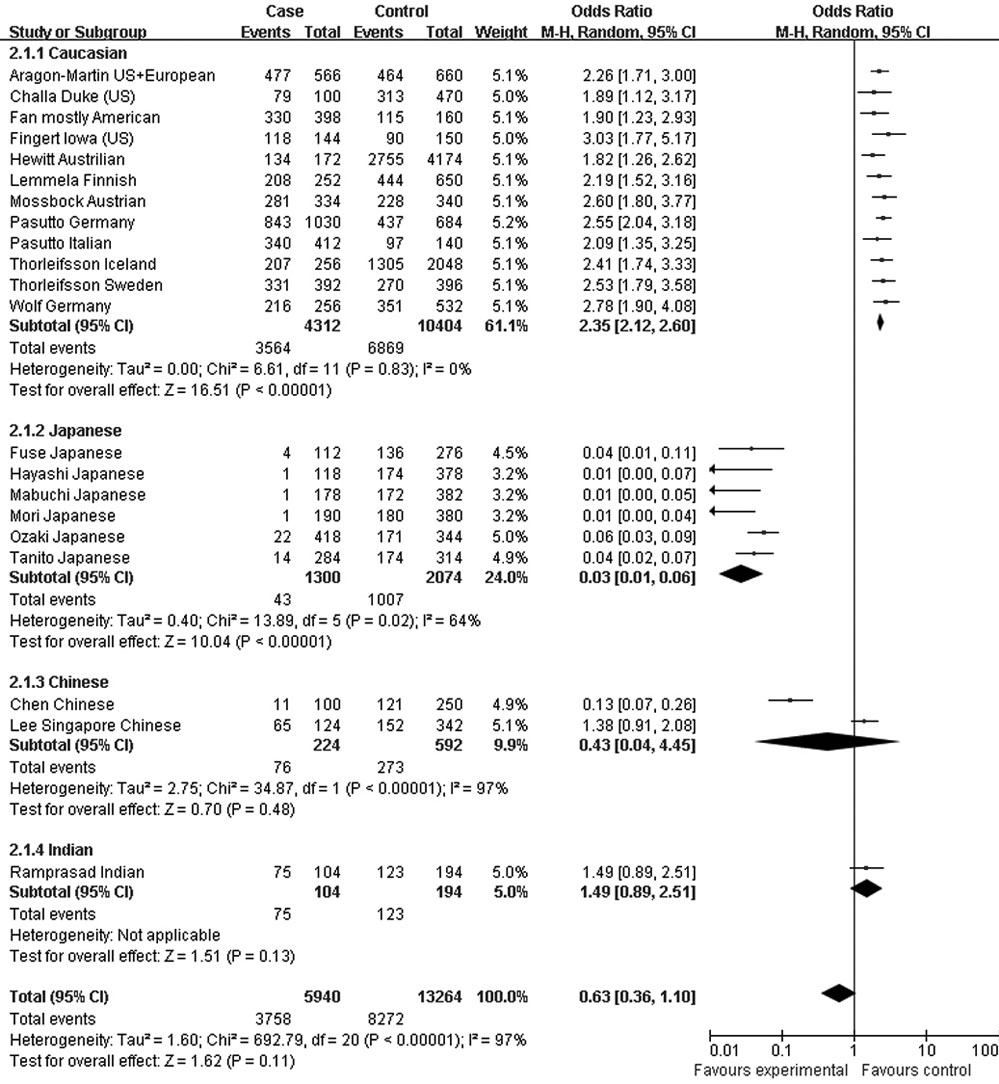
Meta-analysis of the association of single nucleotide polymorphism (SNP) rs1048661 with a combined group of exfoliation syndrome (XFS) and exfoliation glaucoma (XFG). Squares indicate study-specific odds ratio (OR); the size of the box is proportional to the weight of the study; horizontal lines indicate 95% confidence interval (CI); diamond indicates summary OR with its corresponding 95% CI. Subgroup meta-analysis indicated that the ORs of SNP rs1048661 G allele are reversed in Caucasian and Japanese.

**Figure 5:**
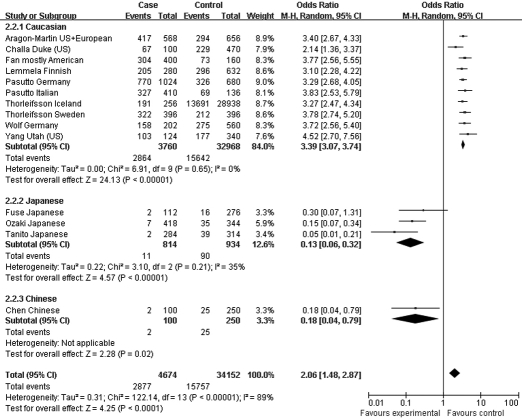
Meta-analysis of the association of single nucleotide polymorphism (SNP) rs2165241 with a combined group of exfoliation syndrome (XFS) and exfoliation glaucoma (XFG). Squares indicate study-specific odds ratio (OR); the size of the box is proportional to the weight of the study; horizontal lines indicate 95% confidence interval (CI); diamond indicates summary OR with its corresponding 95% CI. Subgroup meta-analysis indicated that the ORs of SNP rs2165241 T allele are reversed in Caucasian and Japanese.

**Figure 6:**
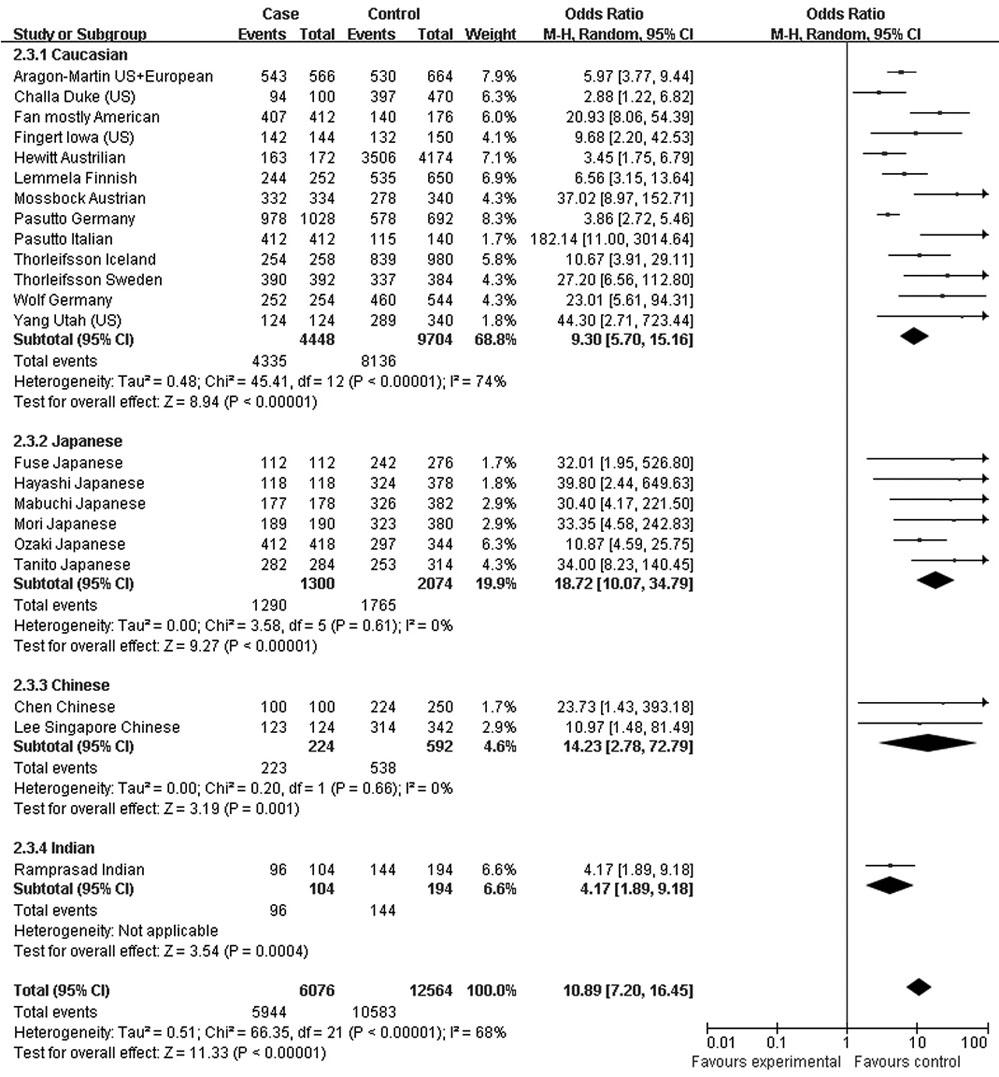
Meta-analysis of the association of single nucleotide polymorphism (SNP) rs3825942 with a combined group of exfoliation syndrome (XFS) and exfoliation glaucoma (XFG). Squares indicate study-specific odds ratio (OR); the size of the box is proportional to the weight of the study; horizontal lines indicate 95% confidence interval (CI); diamond indicates summary OR with its corresponding 95% CI. Subgroup meta-analysis indicated that the ORs SNP rs3825942 G allele is consistent in Caucasian and Japanese.

Moreover, the association profiles of *LOXL1* SNPs were examined by comparing the patients with XFS or XFG versus control subjects in different populations. The study cohorts were from four major ethnic groups: Caucasian, Japanese, Chinese, and Indian. In most individual studies, significant allelic association was found between XFS/XFG and the *LOXL1* SNPs rs1048661, rs2165241, and rs3825942, regardless of ethnicity. In the association of rs3825942 with XFS/XFG, the ORs of the G allele in every study was significantly greater than 1 and the ORs lay on the same side of the *y*-axis. The OR was 9.30 (95% CI 5.70–15.16, p<0.00001) in Caucasian, 18.72 (95% CI 10.07–34.79, p<0.00001) in Japanese, 14.23 (95% CI 2.78–72.79, p=0.001) in Chinese, and 4.17 (95% CI 7.04–16.55, p=0.0004) in Indian populations. The overall OR was 10.89 (95% CI 7.20–16.45, p<0.00001) for the four ethnic populations (Figure 6). Notably, however, the ORs of rs2165241 in Caucasian and Japanese populations were reversed. OR for the T allele was 3.39 (95% CI 3.07–3.74, p<0.00001) in Caucasian populations, suggesting that the T allele was the at-risk allele. The OR for the T allele was 0.13 (95% CI 0.06–0.32, p<0.00001) in Japanese populations, indicating that the T allele was a protective allele (Figure 5). Such discrepancy was also observed in rs1048661, with OR for the G allele being 2.35 (95% CI 2.12–2.60, p<0.00001) in Caucasian, and 0.03 (95% CI 0.01–0.06, p<0.00001) in Japanese populations but not significant in Chinese and Indian populations (Figure 4). Sensitivity tests were subsequently performed. Cohorts whose allele counting data were calculated from the frequencies data, Challa’s Duke (US) cohort [13] and Hewitt’s Austrian cohort [17], were removed. The OR for the rs3825942 G allele was 9.30 and 12.40 in Caucasians before and after removing the two cohorts, respectively. The OR for the rs1048661 G allele was 2.35 and 2.41 in Caucasians before and after removing the two cohorts, respectively. The OR for the rs2165241 T allele was 3.39 and 3.47 in Caucasians before and after removing the two cohorts, respectively.

**Figure 7:**
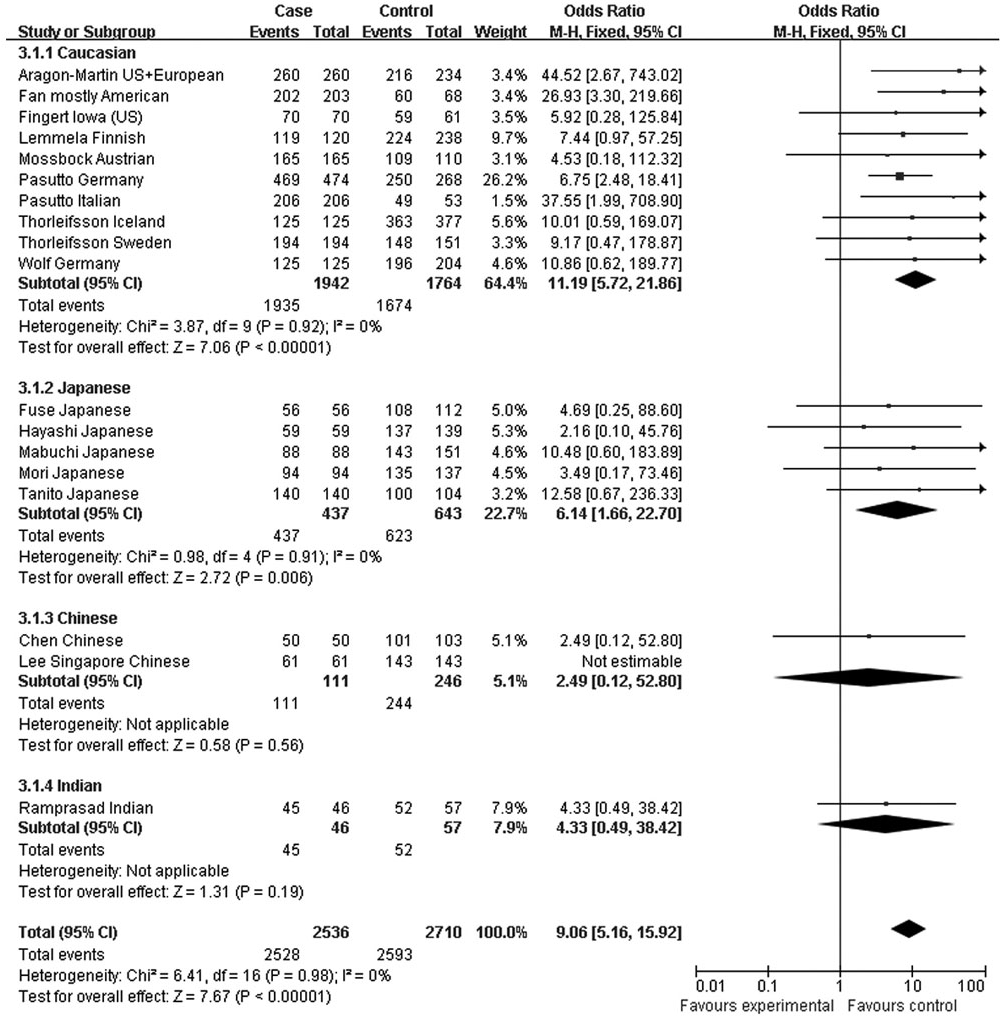
Meta-analysis of the association of single nucleotide polymorphism (SNP) rs3825942 with a combined group of exfoliation syndrome (XFS) and exfoliation glaucoma (XFG) in additive models. Squares indicate study-specific odds ratio (OR); the size of the box is proportional to the weight of the study; horizontal lines indicate 95% confidence interval (CI); diamond indicates summary OR with its corresponding 95% CI. Synthesized homozygous OR (GG versus AA) of SNP rs3825942 was 9.06 for XFS/XFG.

**Figure 8:**
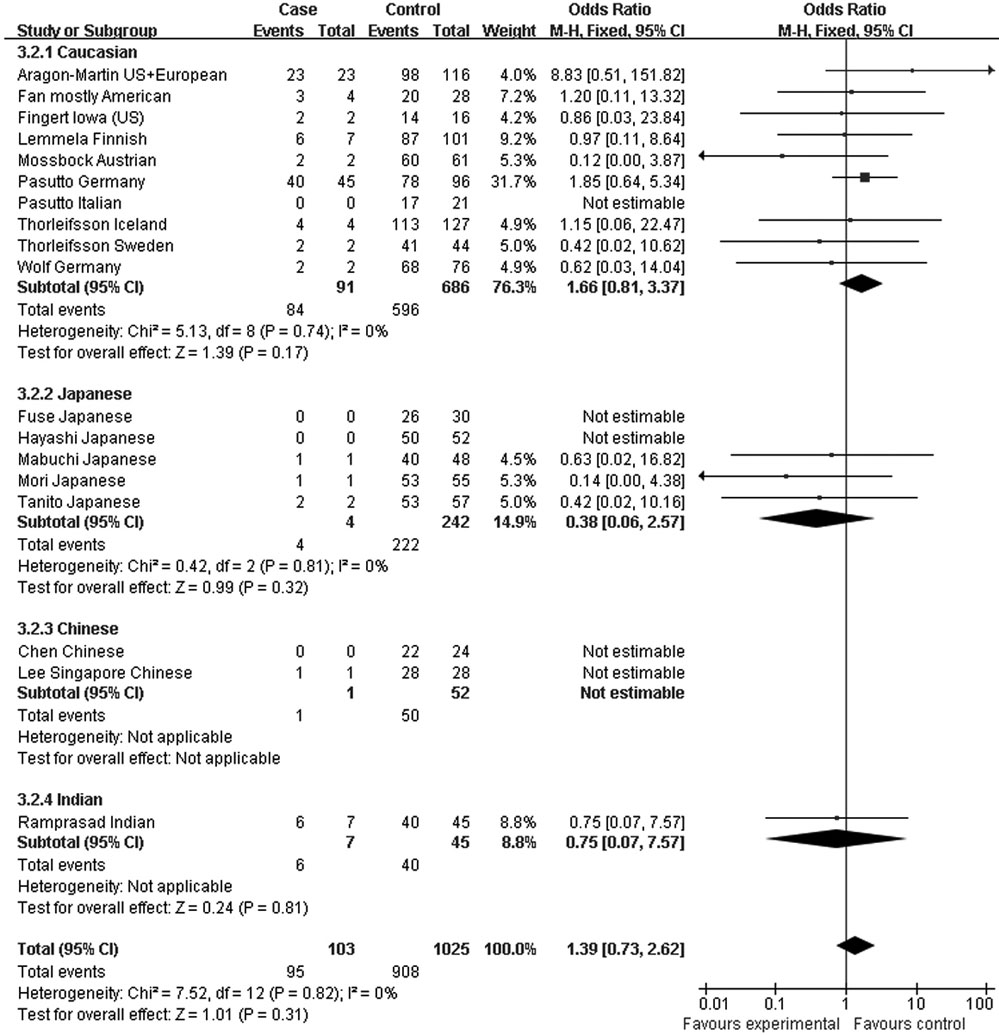
Meta-analysis of the association of single nucleotide polymorphism (SNP) rs3825942 with a combined group of exfoliation syndrome (XFS) and exfoliation glaucoma (XFG) in additive models. Squares indicate study-specific odds ratio (OR); the size of the box is proportional to the weight of the study; horizontal lines indicate 95% confidence interval (CI); diamond indicates summary OR with its corresponding 95% CI. Meta-analysis indicated that the heterozygote genotype (GA) does have higher susceptibility to XFS/XFG compared to AA genotype. **C**: Synthesized OR of SNP rs3825942 was 7.05 for XFS/XFG with dominant model (GG+GA versus AA).

**Figure 9:**
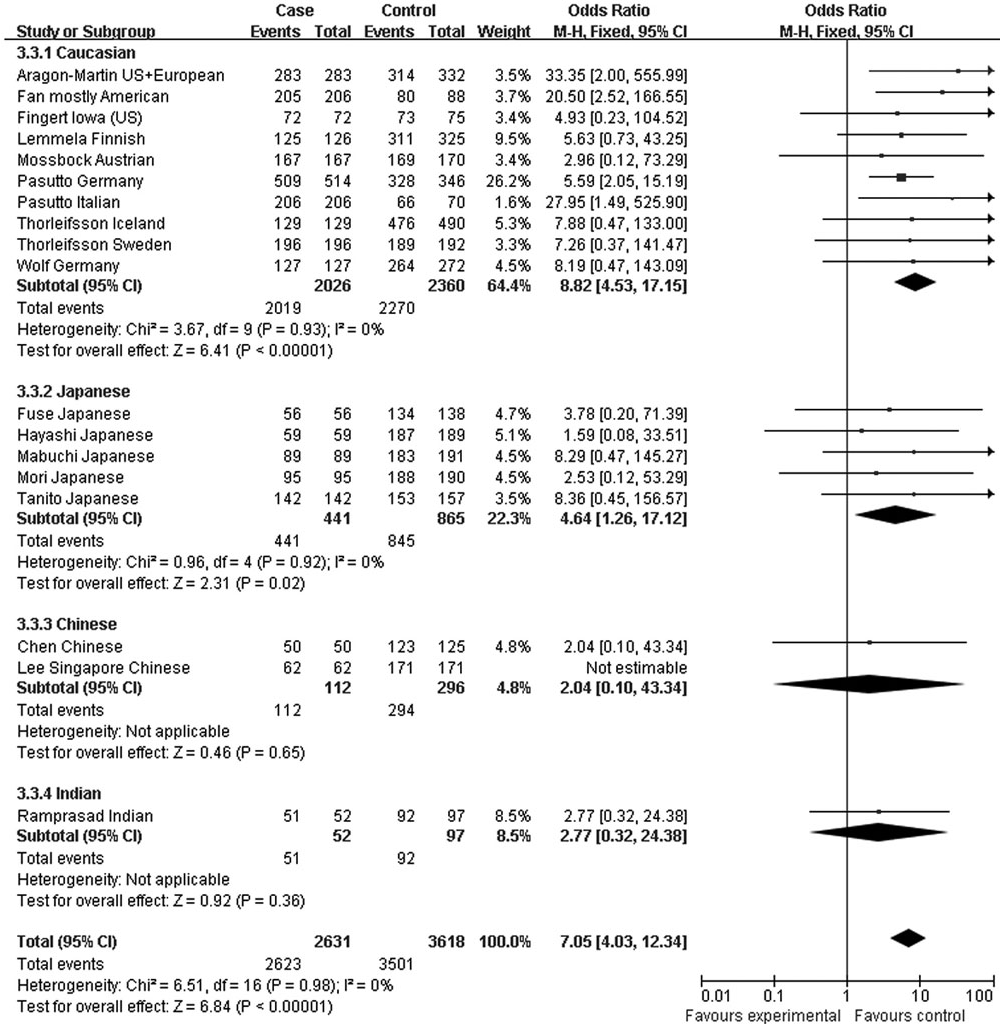
Meta-analysis of the association of single nucleotide polymorphism (SNP) rs3825942 with a combined group of exfoliation syndrome (XFS) and exfoliation glaucoma (XFG) in dominant models. Squares indicate study-specific odds ratio (OR); the size of the box is proportional to the weight of the study; horizontal lines indicate 95% confidence interval (CI); diamond indicates summary OR with its corresponding 95% CI. Synthesized OR of SNP rs3825942 was 7.05 for XFS/XFG with dominant model (GG+GA versus AA).

**Figure 10:**
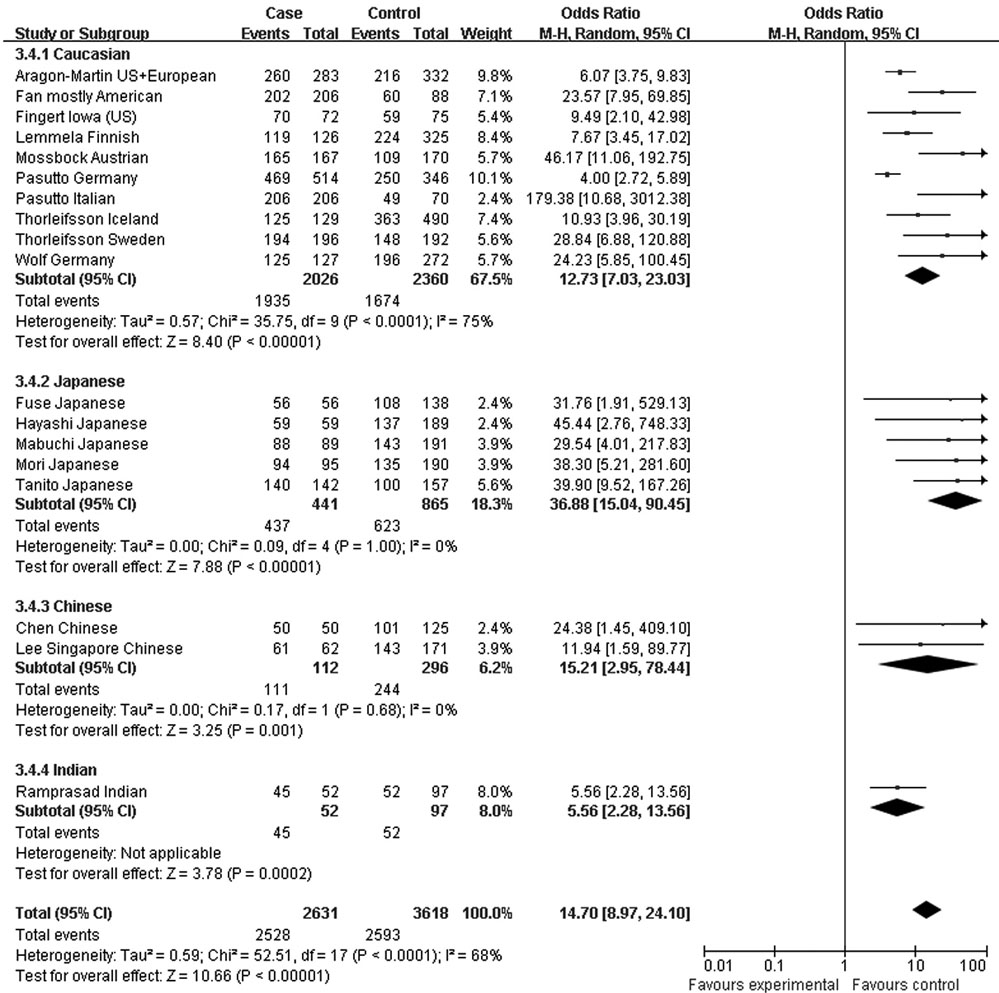
Meta-analysis of the association of single nucleotide polymorphism (SNP) rs3825942 with a combined group of exfoliation syndrome (XFS) and exfoliation glaucoma (XFG) in recessive models. Squares indicate study-specific odds ratio (OR); the size of the box is proportional to the weight of the study; horizontal lines indicate 95% confidence interval (CI); diamond indicates summary OR with its corresponding 95% CI. Synthesized OR of SNP rs3825942 was 14.70 for XFS/XFG with recessive model (GG versus AA+GA).

Since the associations of rs1048661 and rs2165241 are inconsistently reported between Caucasian and Japanese populations, we performed genotype association analysis using different hereditary models only for rs3825942. When using a multiplicative/additive model, the total OR for the homozygous at-risk genotype (GG versus AA) was 9.06 (95% CI 5.16–15.92, p<0.00001, Figure 7), whereas the total OR for the heterozygous genotype (GA versus AA) was not significantly different from 1 (total OR=1.39, 95% CI 0.73–2.62, p=0.31, Figure 8). On the other hand, the total OR was 7.05 (95% CI 4.03–12.34, p<0.00001, Figure 9) under the dominant model and 14.70 (95% CI 8.97–24.20, p<0.00001, Figure 10) under a recessive model. By subgroup-based meta-analysis, the ORs in different ethnic groups were comparable as the 95% CIs partially overlapped.

**Figure 11:**
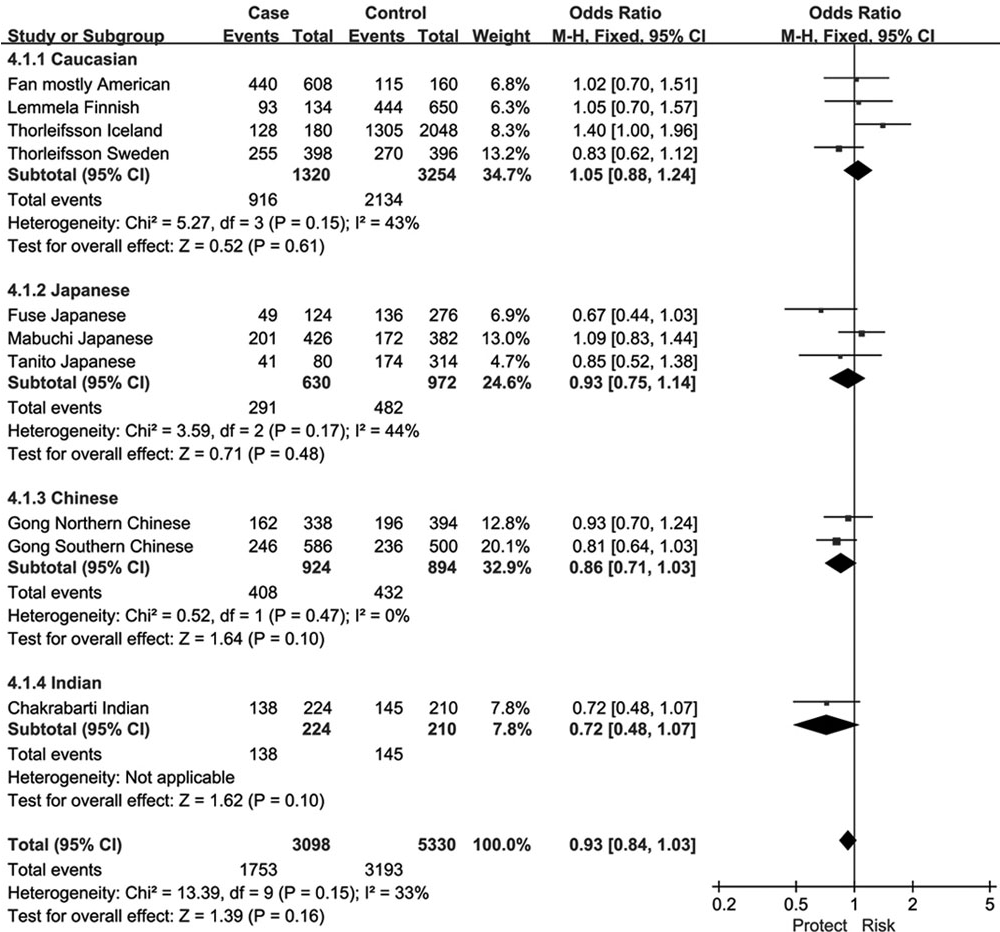
Meta-analysis of the association of single nucleotide polymorphism (SNP) rs1048661 with primary open angle glaucoma (POAG). Squares indicate study-specific odds ratio (OR); the size of the box is proportional to the weight of the study; horizontal lines indicate 95% confidence interval (CI); diamond indicates summary OR with its corresponding 95% CI. Meta-analysis indicated there is no statistical significant association of SNP rs1048661 with POAG.

**Figure 12:**
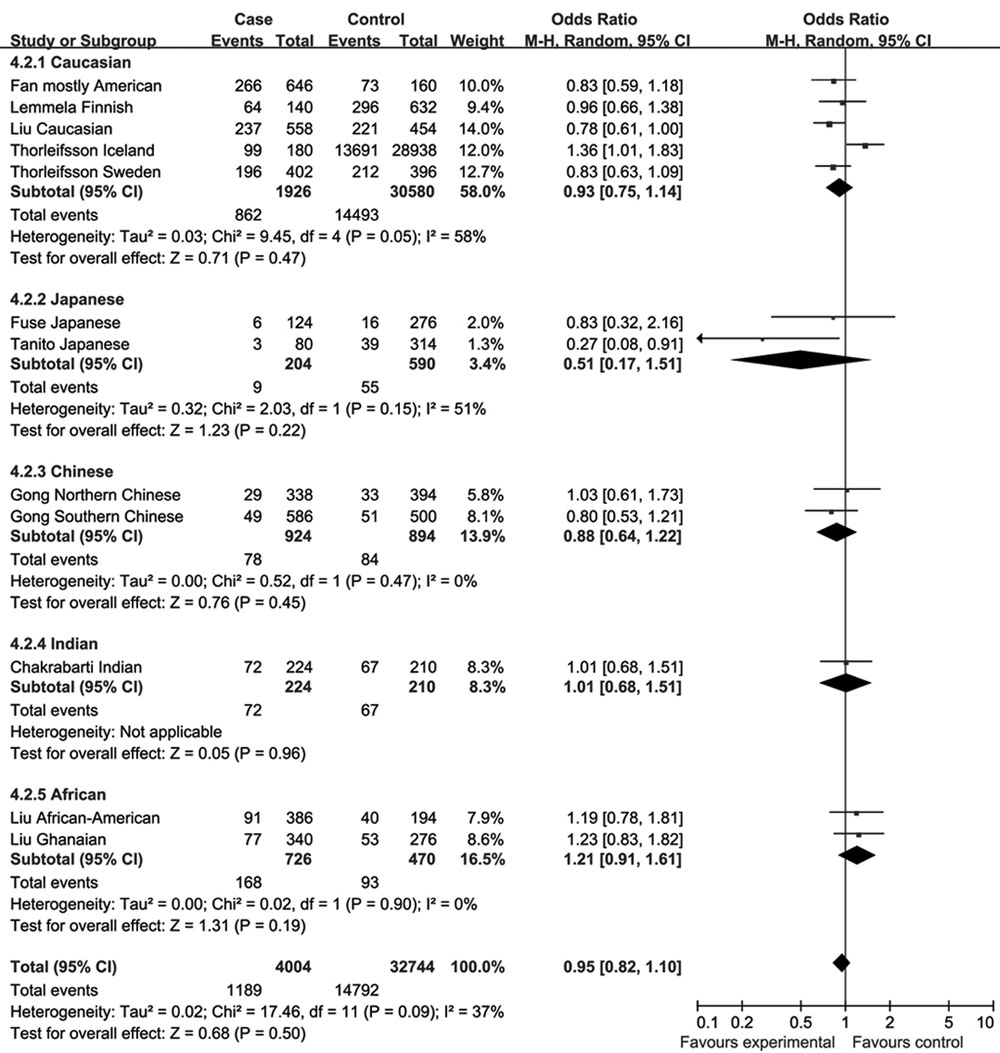
Meta-analysis of the association of single nucleotide polymorphism (SNP) rs2165241 with primary open angle glaucoma (POAG). Squares indicate study-specific odds ratio (OR); the size of the box is proportional to the weight of the study; horizontal lines indicate 95% confidence interval (CI); diamond indicates summary OR with its corresponding 95% CI. Meta-analysis indicated there is no statistical significant association of SNP rs2165241 with POAG.

**Figure 13:**
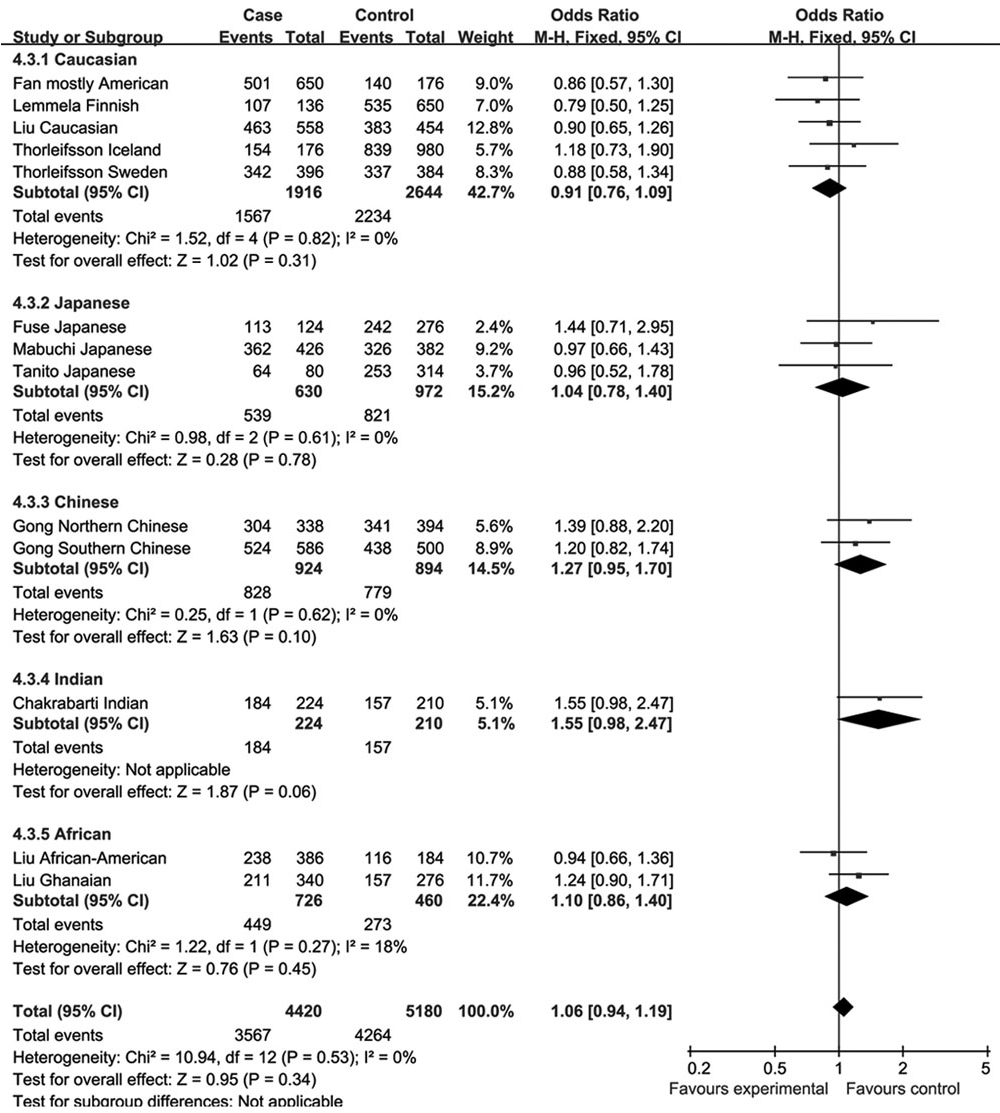
Meta-analysis of the association of single nucleotide polymorphism (SNP) rs3825942 with primary open angle glaucoma (POAG). Squares indicate study-specific odds ratio (OR); the size of the box is proportional to the weight of the study; horizontal lines indicate 95% confidence interval (CI); diamond indicates summary OR with its corresponding 95% CI. Meta-analysis indicated there is no statistical significant association of SNP rs3825942 with POAG.

There was no single article reporting a significant difference in allele distributions of rs1048661 and rs3825942 between POAG patients and control subjects. Our meta-analysis also identified no significant association between SNP rs1048661 or rs3825942 and POAG in any subgroup or the entire study populations. The overall OR was 0.93 (95% CI 0.84–1.03, p=0.16) for the G allele of rs1048661 (Figure 11) and 1.06 (95% CI 0.94–1.19, p=0.34) for the G allele of rs3825942 (Figure 13). By contrast, two out of the 12 cohorts reported significant association between rs2165241 and POAG in Caucasian and Japanese populations. However, their ORs were in opposite directions, and the overall OR of the 12 studies was 0.95 (95% CI 0.82–1.10, p=0.50) for the T allele of rs2165241 (Figure 12). Sensitivity tests were performed in that the cohorts whose allele counting data were calculated from frequency data, Liu’s Caucasian and African cohorts [31] and Chakrabarti’s Indian cohort [33], were removed; however, the results were similar. None of the overall ORs were significantly different from 1 without the two cohorts. Since there was no significant allelic association between the three SNPs and POAG, no further meta-analysis (e.g., genotypic association) was performed.

Apart from XFS/XFG and POAG, there was also one study reporting the *LOXL1* SNPs in primary angle closure glaucoma (PACG), two in normal tension glaucoma (NTG), and two in pigment dispersion syndrome and pigmentary glaucoma. However, none of these studies identified a significant association between the *LOXL1* SNPs and these subtypes of glaucoma (Table 3). In our meta-analysis to investigate the association of *LOXL1* SNPs with NTG and PG, no statistical significant association was found (all p>0.05).

**Table 3:** Characters of reported cohorts of other ocular diseases association with *LOXL1* SNPs.

**First author**	**Cohort**	**Disease**	**Sample size**		**Age (years±SD)**		**Sex (Male %)**		rs1048661 ** G**		rs2165241 ** T**		rs3825942 ** G**		**Ref**
			**Case**	**Control**	**Case**	**Control**	**Case**	**Control**	**Case**	**Control**	**Case**	**Control**	**Case**	**Control**	
Chbakrabarti	Indian	PACG	96	105	NA	NA	NA	NA	65.6	69.0	30.2	31.9	76.6	74.8	[33]
Tanito	Japanese	NTG	54	157	78.3±4.8	77.2±5.1	33.3	28.7	53.7	55.4	13.9	12.4	79.6	80.6	[27]
Wolf	Germany	NTG	273	280	63.9±14.2	66±13	35	41	65.3	66.0	48.9	49.1	85.3	84.6	[19]
Wolf	Germany	PG	88	280	53.8±13.5	66±13	72	41	63.1	66.0	48.8	49.1	88.1	84.6	[19]
Rao	American	PG	44	108	NA	NA	NA	NA	67.9	72.4	52.4	47.1	86.6	82.2	[34]
Rao	American	PDS	34	108	NA	NA	NA	NA	66.7	72.4	50.0	47.1	83.3	82.2	[34]

NA: data not available. Ref: references. NTG: normal tension glaucoma. PACG: primary angle closure glaucoma; PG: pigmentary glaucoma. PDS: pigment dispersion syndrome.

## Discussion

Exfoliation glaucoma (XFG), a consequence of exfoliation syndrome (XFS), is the most common form of secondary open angle glaucoma. Approximately 25% patients with XFS present with increased IOP, and one-third have developed glaucoma [19]. To date, association of SNPs in the *LOXL1* gene with XFS and/or XFG has been shown across different populations; however, there were several different phenotypes in these studies. Thus, it is necessary to test whether XFS and XFG are of genetic homogeneity. In the first part of this present meta-analysis, the distribution profiles of the *LOXL1* SNPs between XFS and XFG were evaluated. By reviewing all the reported association profiles with meta-analysis, no statistical difference in any population group or overall study subjects was identified for any of the three SNPs (rs1048661, rs2165241, and rs3825942). All the p values were >0.05.

Since *LOXL1* SNPs are not heterogenic in XFS and XFG, we combined these two phenotypes in our analysis. Our results also suggested that the *LOXL1* gene may contribute to disease onset of the exfoliation disease rather than just increased IOP. However, the current meta-analysis cannot prove this hypothesis because the data are from retrospective case-control studies. Further prospective longitudinal studies are warranted.

In most reported studies on *LOXL1* and XFS/XFG, the three SNPs (rs1048661, rs2165241, and rs3825942) have been investigated together [11-23,25-28]. They are in strong LD among different populations. The haplotype defined by these SNPs was also significantly associated with the disease [11,13,20,26,28]. So far, however, there is no consolidated evidence to show which SNP plays a more major role in the molecular pathogenesis of XFS/XFG. On the other hand, the allelic and genotypic distributions of the three SNPs were found to be drastically different among different populations [11-23,25-28]. Genetic diversity occurs across different ethnicities [35,36]. Therefore, we resolved to find out which SNP plays a role across different populations. In the first part of our meta-analysis, we identified genetic homogeneity of the *LOXL1* SNPs between XFS and XFG. Therefore, the two disease groups were combined in the second part of the meta-analysis of genetic association in subpopulations. We found that the distribution was similar within each individual ethnic group for all three SNPs, rs1048661, rs2165241, and rs3825942. However, the allelic distributions of Japanese and Caucasian populations are reversed for rs1048661 and rs2165241. The T allele of rs2165241 and the G allele of rs1048661 are the at-risk alleles in Caucasians, with an OR of 3.39 and 2.35, respectively. In contrast, the two alleles are protective in the Japanese population, with an OR of 0.13 and 0.03, respectively. All the ORs are statistically significant with p values <0.00001. Therefore, it is more likely that SNPs rs1048661 and rs2165241 are not directly implicated in the pathogenesis of XFS/XFG. In contrast, the distribution of SNP rs3825942 followed a similar pattern in all three ethnic groups. The G allele was the at-risk allele, and the OR was 9.30, 18.72, 10.97, and 4.17 in Caucasian, Japanese, Chinese and Indian populations, respectively. The total OR was 10.75 (95% CI 7.08–16.31). Our finding suggests that rs3825942 is the common disease-associated polymorphism across different populations and may have functional impacts on the LOXL1 protein and contribute to the pathogenesis of XFS/XFG. Moreover, in the third part of this meta-analysis, we found that the OR for the homozygous genotype of rs3825942 is 9.42, while the OR for the heterozygote is not statistically significant. Moreover, the OR in the recessive model was the highest among different genetic models (OR=14.55). Therefore, it could be inferred that rs3825942 plays a role in a recessive pattern; however, what functional role rs3825942 played in the pathogenesis of XFS/XFG remains unclear. *LOXL1* is located in 15q22, encoding a member of the lysyl oxidase family. The LOXL family is an extracellular copper-dependent amine oxidase that involved in the first step of the formation of cross-links in collagen and elastin. Therefore, sequence variation in *LOXL1* may influence the function, synthesis, and subsequent deposition of the extracellular tissues [37]. SNP rs3825942 is located in the first exon of *LOXL1* and leads to a nonsynonymous amino acid change from glycine to asparagine at position 153 (G153D). However, functional prediction using in silico programs Polymorphism Phenotyping (PolyPhen) and Sorting Intolerant From Tolerant (SIFT) suggested that the amino acid substitution is benign and tolerated (data not shown). The exact functional effects of this substitution remain to be further investigated.

The association of *LOXL1* SNPs with other types of glaucoma, including POAG, NTG, pigmentary glaucoma, and PACG, were reviewed in this meta-analysis. However, no significant association was found between POAG and *LXOL1* SNPs after merging the results from 13 cohorts. Therefore, it is likely that *LOXL1* is not implicated in the primary open angle glaucomatous mechanism. Associations between *LXOL1* SNPs and NTG, pigmentary glaucoma, or PACG were also negative, although the number of articles included in this meta-analysis was limited. The lack of association between *LOXL1* and primary glaucoma has provided evidence supporting that *LOXL1* is linked to the pathogenesis of the exfoliation syndrome but not the direct genetic cause of IOP elevation and subsequent glaucoma.

In summary, by using meta-analysis the genetic homogeneity of *LOXL1* between XFS and XFG has been confirmed. The genetic effect of rs3825942 is similar in different populations in a recessive genetic model. We detected inconsistencies in the effect of rs1048661 and rs2165241 between Caucasian and Japanese populations. Our results also revealed that the *LOXL1* gene is not a susceptibility gene of other types of glaucoma other than XFG. Further genetic studies are required to unravel the discrepancy in LD patterns of the *LOXL1* gene among different populations.
